# Effects of Multimodal AR-HUD Navigation Prompt Mode and Timing on Driving Behavior

**DOI:** 10.3390/jemr18060063

**Published:** 2025-11-04

**Authors:** Qi Zhu, Ziqi Liu, Youlan Li, Jung Euitay

**Affiliations:** 1 School of Packaging Design and Arts, Hunan University of Technology, Zhuzhou 412007, China; kikichul621@hanyang.ac.kr; 2 Graduate School, College of Design, Hanyang University, Seoul 04763, Republic of Korea; liyoulan0716@hanyang.ac.kr; 3 School of Art and Design, Hubei University of Technology, Wuhan 430068, China; liuziqi647@hbut.edu.cn; 4 Department of Communication Design, College of Design, Hanyang University ERICA, Ansan 15588, Republic of Korea

**Keywords:** prompt timing, multimodal, AR-HUD, continuous auditory prompt, visual navigation, eye movement, driving behavior

## Abstract

Current research on multimodal AR-HUD navigation systems primarily focuses on the presentation forms of auditory and visual information, yet the effects of synchrony between auditory and visual prompts as well as prompt timing on driving behavior and attention mechanisms remain insufficiently explored. This study employed a 2 (prompt mode: synchronous vs. asynchronous) × 3 (prompt timing: −2000 m, −1000 m, −500 m) within-subject experimental design to assess the impact of multimodal prompt synchrony and prompt distance on drivers’ reaction time, sustained attention, and eye movement behaviors, including average fixation duration and fixation count. Behavioral data demonstrated that both prompt mode and prompt timing significantly influenced drivers’ response performance (indexed by reaction time) and attention stability, with synchronous prompts at −1000 m yielding optimal performance. Eye-tracking results further revealed that synchronous prompts significantly enhanced fixation stability and reduced visual load, indicating more efficient information integration. Therefore, prompt mode and prompt timing significantly affect drivers’ perceptual processing and operational performance. Delivering synchronous auditory and visual prompts at −1000 m achieves an optimal balance between information timeliness and multimodal integration. This study recommends the following: (1) maintaining temporal consistency in multimodal prompts to facilitate perceptual integration and (2) controlling prompt distance within an intermediate range (−1000 m) to optimize the perception–action window, thereby improving the safety and efficiency of AR-HUD navigation systems.

## 1. Introduction

Augmented reality head-up displays (AR-HUDs) significantly enhance drivers’ information acquisition efficiency and spatial perception by integrating navigation information with the road environment [1]. However, as visual elements in AR-HUDs become increasingly rich, drivers’ reliance on visual navigation intensifies, which may cause attentional distraction and sensory overload, thereby affecting driving behavior stability [2]. To alleviate the burden on the visual channel, auditory prompts, as an important non-visual channel complement, have been gradually integrated into multimodal AR-HUD navigation systems [3]. The effectiveness of multimodal interaction does not depend solely on the number of information sources but rather on the dynamic coordination of temporal rhythm and semantic logic among different channels [4]. Particularly, in navigation tasks, temporal discrepancies such as anticipation, delay, or rhythm inconsistency between auditory and visual prompts can weaken the clarity of navigation information, leading to operational delays or erroneous judgments [5,6]. Therefore, systematic investigation of prompt timing optimization, especially the temporal coordination between auditory and visual navigation prompts in multimodal AR-HUD systems, is essential for enhancing driving behavior and improving navigation system reliability.

Driving behavior involves a complex process influenced by various internal and external factors [7]. Within this process, the interface design of AR-HUD navigation systems acts as an external information medium directly affecting drivers, whose perceptual properties significantly influence drivers’ attention allocation and operational responses [8]. Existing studies have shown that color usage in AR-HUD navigation interfaces plays a key role in visual prioritization, but overly saturated or high-contrast colors may induce interference effects [9,10]. Moreover, the size and layout of visual icons have been confirmed to relate closely to attention, cognitive load, and driving experience [11,12]. Beyond static design elements, dynamic components such as animated prompts can enhance perceptual salience but may also cause attentional shifts or delayed responses under high-workload conditions [13]. Apart from vision, although limited in quantity, studies have proposed augmented reality navigation systems combining audio and haptic feedback (HapAR) to improve spatial perception and mobility safety for visually impaired users [14]. Despite the widespread attention to visual prompts in AR-HUD systems, most research focuses on interface design style, visual clarity, and visual attention allocation within a single modality. In actual driving scenarios, however, visual navigation often coexists with auditory prompts and even haptic vibrations. The existing literature lacks systematic exploration of how visual navigation integrates with other modalities in AR-HUD systems. Thus, further research on the collaborative design of visual and other modality prompts in AR-HUD navigation will facilitate intelligent upgrades and the human factors optimization of navigation systems.

Prompt timing control has been demonstrated as a critical factor influencing driving behavior [15]. Both premature and delayed prompts can disrupt drivers’ perceptual rhythm and operational planning [16]. Early prompts risk being forgotten before execution, leading to deviations or secondary confirmation behaviors [17]. For example, providing a right-turn prompt at 2000 m may be forgotten due to traffic changes or attentional shifts, necessitating rechecking of the navigation. Conversely, late prompts force drivers to make decisions and respond within limited time, significantly increasing operational stress and traffic risks [18]. For instance, issuing a right-turn prompt only 200 m from the intersection leaves insufficient time for lane changes, deceleration, or traffic assessment.

In addition to timing, prompt mode also significantly impacts driving behavior [19], especially in AR-HUD systems. Visual prompts usually overlay AR images in the forward field of view, while auditory prompts are delivered via the auditory channel. A lack of temporal coordination between these two modalities can cause rhythm mismatches, interrupting drivers’ information processing and response flow [20]. According to [21], multimodal cues presented simultaneously are considered temporally synchronous, which affects sensory information integration across modalities. Conversely, temporal asynchrony typically leads to separate object interpretations. The authors in [22] also confirmed that synchronous multimodal cues form a unified object interpretation, while asynchronous presentation causes dual-object interpretation. Temporally asynchronous prompts present different modalities in sequence, e.g., first an auditory “Right turn in 500 m” followed by a visual right-turn arrow on the AR-HUD. This reduces instantaneous information load but may increase crossmodal integration burden. Synchronous prompts present auditory and visual information simultaneously, e.g., the AR-HUD displays a right-turn arrow while the auditory prompt “Right turn in 500 m” is played, reducing cognitive conflicts caused by temporal inconsistency but potentially increasing instantaneous cognitive load in information-dense scenarios. This study designs two prompt mode conditions (synchronous vs. asynchronous) to examine the differential effects of auditory–visual navigation prompt matching on driving behavior and visual attention allocation.

In summary, although progress has been made in interface visual design, modal information integration, and prompt content optimization, the exploration of multimodal prompt mode and timing remains insufficient. When and in what sequence auditory and visual prompts appear directly affect the information integration efficiency driving behavior stability. However, the existing literature has yet to reach consensus on the effects of synchronous versus asynchronous presentation, and systematic research on this topic in AR-HUD systems is lacking. Therefore, this study, based on a virtual driving simulation platform and employing two-way ANOVA with prompt mode (synchronous vs. asynchronous) and prompt timing (−2000 m, −1000 m, −500 m) as independent variables, systematically evaluates the comprehensive effects of multimodal navigation prompts under continuous auditory prompt conditions on drivers’ response efficiency (Reaction Time), visual attention allocation (Eye Movement Metrics), and Situation Awareness Rating Technique (SART).

To achieve these objectives, this study focuses on the temporal coordination of auditory and visual prompts under continuous auditory prompt conditions and proposes the following two core research questions (RQ):

- RQ1: Does the prompt mode (synchronous vs. asynchronous) of auditory and visual prompts significantly affect drivers’ operational performance in AR-HUD navigation systems?
- RQ2: Under continuous auditory prompt conditions, which prompt timing (−2000 m, −1000 m, −500 m) optimizes modality integration and navigation efficiency?

To answer RQ1, the study sets synchronous (simultaneous appearance) and asynchronous (visual first, auditory later) conditions for auditory and visual navigation prompts. Behavioral response data and SART scores were collected to assess situation awareness under different prompt mode strategies. To answer RQ2, three prompt timing points (−2000 m, −1000 m, −500 m), representing different distance windows before navigation events, were designed to compare driving behavior differences and explore the superiority of synchronous or asynchronous prompts at each timing.

## 2. Literature Review

Multimodal navigation systems typically integrate multiple sensory channels such as visual, auditory, and haptic modalities to accommodate diverse user perceptual preferences and usage contexts [23]. Ref. [24] noted that different modality combinations can significantly enhance human–computer interaction comfort and efficiency under specific environments. For example, in noisy settings, vibration feedback conveys directional information more effectively than auditory prompts; conversely, audio feedback may be preferable when users seek more detailed information about navigation environments, such as landmarks or traffic signs. Some studies have shown that combined audiovisual modes improve situational awareness and reduce risky driving behaviors compared to purely visual or auditory outputs [25,26]. However, other research found that although audiovisual combinations enhance subjective preference, they do not necessarily improve driving behavior significantly [27]. While researchers have improved subjective preference, prompt type selection, and multimodal matching, the critical variable of prompt timing—i.e., when modality information is presented—has been insufficiently explored. Overly long prompt durations may cause cognitive overload and habituation, leading to driver fatigue and neglect, whereas overly brief prompts can cause inattention or, in severe cases, driving hazards. Therefore, this study focuses on the visual and auditory modalities in AR-HUD navigation systems, systematically investigating their effects on driving behavior and situation awareness under different prompt timing conditions, aiming to optimize multimodal navigation design from a temporal perspective to enhance system practicality and safety.

Previous research has confirmed the advantages of continuous auditory prompts. In continuous auditory prompting, navigation information typically consists of multiple staged instructions, making temporal coordination between modalities increasingly critical [28]. Continuous auditory prompts are usually presented as multiple staged vocal messages [29]. Such continuous vocal instructions help drivers maintain stable attention to route targets and reduce cognitive load accumulation caused by interrupted prompts [29]. Studies have analyzed how the timing and content of continuous auditory prompts in navigation systems affect drivers’ psychological states and operations on urban highways [19]. Existing findings indicate that different types of prompt information at −400 m, −300 m, and −200 m distance points can significantly optimize vehicle performance indicators and improve driving behavior under continuous auditory prompting [30]. Furthermore, Yang et al. (2024) [31] found that four types of navigation prompt information start to take effect approximately 200 m upstream of gate barriers, while differences are significant in the area from 100 m upstream to 100 m downstream of intersection stop lines. These studies collectively validate the importance of prompt timing for driving behavior and emphasize the role of continuous auditory prompts in driving behavior in complex environments. However, most research remains confined to timing corollary within auditory systems, lacking an in-depth exploration of the temporal coordination between auditory and visual modalities in AR-HUD navigation under continuous auditory prompt conditions and how this temporal relationship affects drivers’ information processing efficiency and operational behavior.

In multimodal navigation systems, visual prompts serve as a key information transmission channel, and their design strategies directly impact drivers’ attention allocation and information processing efficiency [32]. Prior studies demonstrate that color plays an important role in visual navigation information, eliciting different emotional responses and cognitive loads among drivers [33]. For instance, red, due to its high saturation and alerting property, is widely used to emphasize urgent or important information, whereas blue exerts a calming effect, helping to reduce driving stress [34,35]. Additionally, dynamic visual elements such as gradient animations effectively guide gaze flow, enhancing prompt saliency and recognition and preventing visual fatigue and distraction caused by static graphics. Related studies have also pointed out the significant advantages of visual gradients in directing drivers’ attention to key navigation information [36,37]. Based on these theoretical and empirical findings, this study selected a blue-to-red gradient design scheme intended to convey the importance and urgency of navigation prompts through color gradients, leveraging the high saliency of red for critical warnings and the calming effect of blue for anticipated information. This dynamic color coding supports visual prioritization and enhances the effectiveness of the visual prompt, operating by clearly signaling distance proximity, thereby indirectly aiding the perceptual integration of visual and auditory information rather than claiming a direct modal synergy with the auditory prompt.

## 3. Methods

### 3.1. Experimental Participants and Equipment

To ensure sufficient statistical power for the experimental design, the required sample size was estimated using G*Power 3.1.9.7 (developed by the University of Düsseldorf, Düsseldorf, Germany). In general, a statistical power of 0.8 or higher is considered robust; therefore, power was typically set to 0.8 [38]. According to [39], an effect size (*f*) of 0.25 represents a medium effect, whereas values above 0.4 indicate a large effect. In academic research, medium or larger effect sizes are generally regarded as having practical significance. In this study, the significance level was set at α = 0.05, and the statistical power was set at 0.95, to ensure the robustness and credibility of the inferences. The calculation results indicated that at least 18 participants would be required to meet the analytical needs. Considering experimental conditions and data quality control, a total of 30 participants were actually recruited and included in the analysis, further enhancing the stability and representatives of the results.

A total of 30 participants were recruited, with an approximately balanced gender distribution. Ages ranged from 18 to 26 years, with a mean age of 22.07 years (SD = 2.43). All participants were right-handed, had normal or corrected-to-normal vision, and possessed a valid driver’s license, with an average driving experience of 2.21 years (SD = 3.11). Prior to participation, all subjects provided written informed consent. Upon completion of the experiment, each received CNY 150 as compensation. This study was approved by the Hanyang University Institutional Review Board and strictly adhered to the ethical principles outlined in the Declaration of Helsinki (revised in 2008).

**Figure 1:**
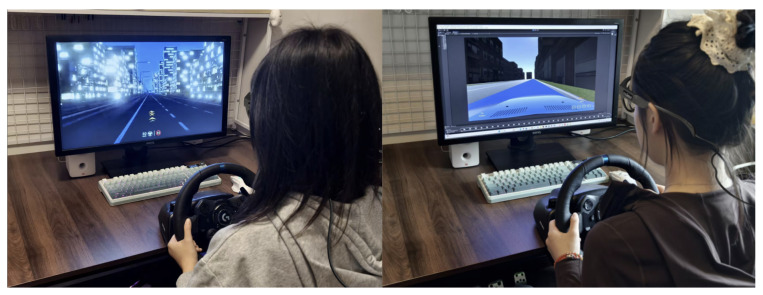
On-site view of the experimental setup.

To ensure experimental controllability and ecological validity, a virtual driving environment was developed using Unity 2022 LTS (developed by Unity Software Inc., San Francisco, CA, USA) with C# scripts used to precisely control auditory and visual prompts. The experimental scene was presented on a 34-inch high-definition monitor (resolution 2560 × 1440, aspect ratio 16:9, dimensions approximately 79 cm × 34 cm), approximating the field of view of a typical vehicle windshield. To enhance driving realism, the setup included a Logitech G29 steering wheel (developed by Logitech International S.A, Lausanne, Switzerland) accelerator and brake pedals, and a standard driver’s seat. The steering wheel buttons were used to record behavioral reaction times. Additionally, participants wore a Tobii Pro Glasses 3 eye tracke (developed by Tobii AB, Stockholm, Sweden)r to collect real-time eye movement data, enabling analysis of attention distribution and visual behavior. As we show at Figure 1.

The image source was placed at a virtual image distance (VID) of 2.5 m. Although ideal AR-HUD systems typically recommend a VID of 10 m to 15 m or longer to minimize vergence–accommodation conflict (VAC) and parallax effects, this simulator configuration utilizes near-field projection, designed to ensure strict control over visual temporal synchronization and clarity, thereby isolating the core effect of multimodal synchrony.

### 3.2. Experimental Design

**Figure 2:**
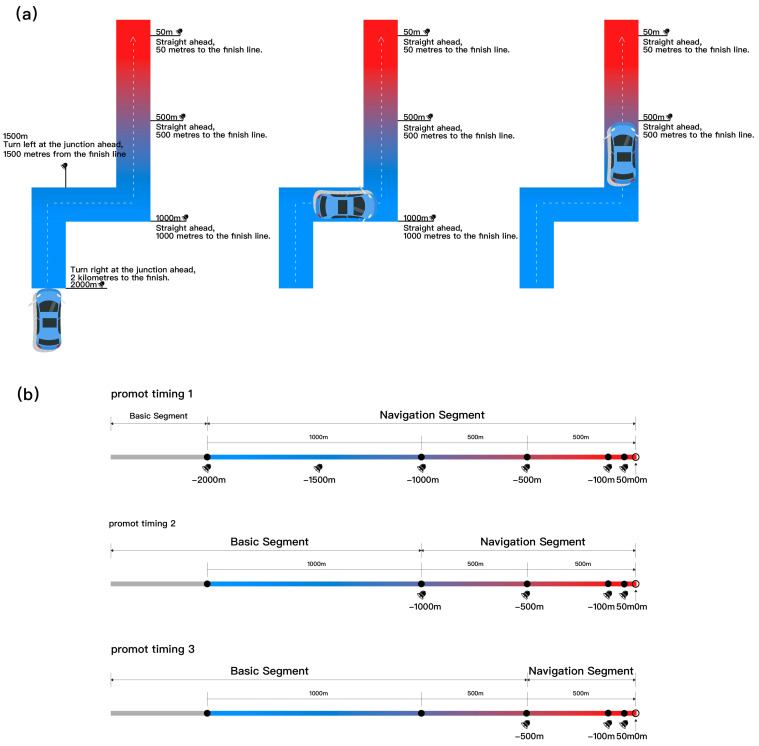
Schematic diagram of the experimental scene and prompt timing settings: (**a**,**b**) show the route scenario and prompt timing under the synchronous condition.

**Figure 3:**
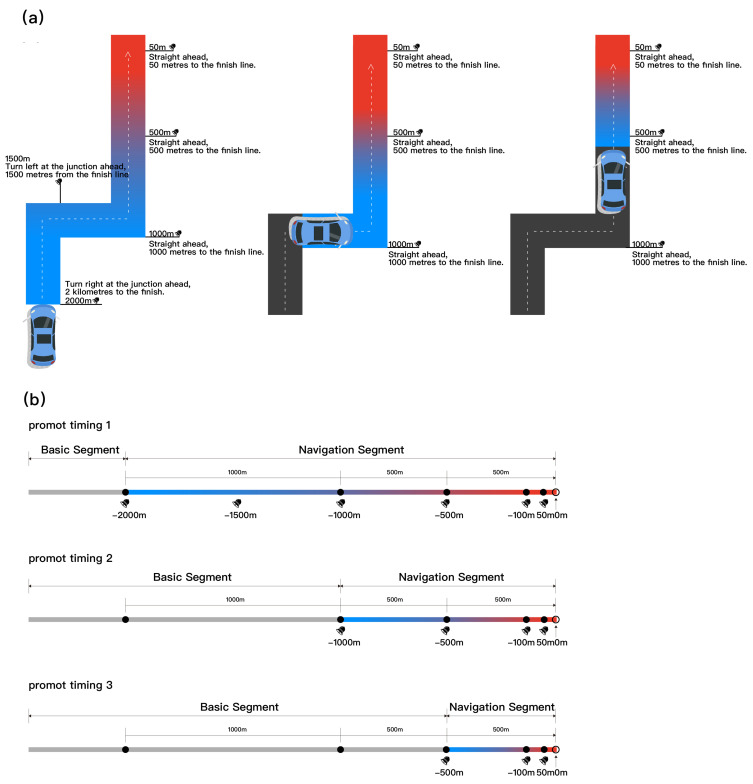
Schematic diagram of the experimental scene and prompt timing settings: (**a**,**b**) show the route scenario and prompt timing under the asynchronous condition. The three prompt lead distances were 2000 m, 1000 m, and 500 m.

The experiment adopted a within-subject design. The original plan comprised a 2 (prompt mode: synchronous vs. asynchronous) × 3 (prompt timing: −2000 m, −1000 m, −500 m) factorial structure, resulting in six conditions. However, pilot testing revealed no substantial difference between the synchronous and asynchronous settings at the 2000 m level, as the visual and auditory stimuli were identical in both cases. Consequently, these two conditions were treated as equivalent in the experiment. To avoid pseudo-independence from repeated measurements and analytical bias, the experimental conditions were treated as five independent conditions for statistical analysis. For clarity, the original factorial matrix is presented in Figure 2 and Figure 3 with a footnote indicating that the 2000 m synchronous/asynchronous conditions were considered equivalent/merged, and only one dataset was retained for analysis. This equivalence occurs because at the early 2000 m warning distance, the prompt serves only as an initial alert function. Regardless of the subsequent asynchronous setup, both modes presented the initial “2000 m turn right” instruction (visual icon and auditory voice) simultaneously. Therefore, at this earliest node, the functional temporal difference between the modes was negligible. In Figure 2 and Figure 3 we present two different types of prompts simultaneously for design illustration. However, the table notes clearly specify that the 2000 m synchronous/asynchronous conditions are treated as equivalent/merged, and only one set of data was retained for analysis.

In the synchronous condition, visual navigation and auditory prompts were presented simultaneously at −2000 m, −1000 m, and −500 m relative to the navigation point. In the asynchronous condition, the visual navigation remained continuously present, whereas the auditory prompts appeared independently at the above-three nodes, simulating a real-world delayed voice prompt mode. The aim of this study was to evaluate their combined effects on drivers’ situation awareness, driving stability, and subjective experience. The system recorded reaction times, subjective situation awareness ratings, and other metrics to comprehensively assess the impact of multimodal prompt timing on driving behavior and user experience.

### 3.3. Experimental Materials

The core of this study was to construct temporal alignment patterns between auditory and visual prompts to simulate multimodal prompt strategies in an AR-HUD navigation system. Visual prompts consisted of dynamic gradient color bars combined with directional arrows, which were overlaid in augmented reality onto the driver’s field of view to simulate the actual display effect of an AR-HUD. The gradient color transitioned from blue to red as the navigation node approached, aiming to enhance visual hierarchy and improve recognizability and attention capture efficiency [40]. The auditory prompts consisted of continuous natural voice instructions delivered through headphones, providing explicit navigation guidance, such as “You will arrive at your destination in 1000 m.” The voice prompts used in this experiment were completely standardized, only providing information about the remaining distance and directional cues, in order to avoid any effects caused by inconsistencies in auditory information.

All experimental materials were developed in Unity to realistically simulate a first-person driving environment. The Unity scene was based on a standard urban arterial road model, approximately 5 km in length, containing typical traffic elements such as lane markings, buildings, and roadside greenery to create an ecologically valid driving environment. The ego vehicle traveled at a constant speed along the road. Three key navigation nodes were pre-set on the main road, located approximately −2000 m, −1000 m, and −500 m from the turning point, to trigger the auditory and visual prompts. Ensuring that the range from −2000 m to −500 m covered the early anticipation, mid-course adjustment, and final execution stages of navigation. This design ensured both practical alignment with real-world navigation habits and a maximized temporal separation between prompt modes. The experiment then collected both objective and subjective data from participants. Figure 1 shows a real-world photo of the experiment in progress, and Figure 2 and Figure 3 presents the schematic diagram of the experimental scene and equipment.

### 3.4. Data Collection and Analysis

To systematically evaluate the effects of prompt mode and prompt timing in AR-HUD navigation on drivers’ reaction time and attention allocation, this study adopted a combined objective–subjective assessment approach. Objective measures included reaction time and eye-tracking data, while the subjective dimension was assessed using the Situation Awareness Rating Technique (SART). Following the commonly used reaction-time measurement methods in previous studies, drivers’ reactions to prompts were determined by measuring the time taken to press the brake pedal. Therefore, in this study, eye-tracking metrics were employed to evaluate participants’ performance under different prompt conditions, allowing for a comprehensive assessment of the perceptual efficiency of AR-HUD navigation information and the allocation of attentional resources. Eye-tracking indicators can, to a certain extent, reflect the degree of attention drivers devote to navigation information and the distribution of cognitive resources during the driving task.

In the eye-tracking analysis, the AR-HUD navigation graphical interface was defined as the Area of Interest (AOI), with two core metrics examined: average fixation duration (AFD) and fixation count (FC) [41]. AFD represents the cumulative visual attention time invested by drivers in the AOI and is generally positively associated with information complexity, cognitive processing demand, or interface attractiveness. Short fixation durations (400–600 ms) typically indicate that navigation icons are clearly designed and easy to interpret, whereas excessively long fixations (>600 ms) may reflect complex information or increased cognitive load. FC represents the frequency of gaze within the AOI; higher counts may suggest greater task engagement or stronger information-processing demands. An FC of 1–3 was considered indicative of low attention, suggesting that the prompt did not capture the driver’s focus; 4–6 was regarded as optimal; and 7 or more may indicate redundant information processing or repeated allocation of attentional resources.

Furthermore, to capture participants’ subjective experiences, this study employed an extended version of the SART scale to assess situation awareness (SA) during the experiment. The scale was based on the three core dimensions proposed by [42]: supply, demand, and clarity. Supply assesses whether participants have sufficient attentional resources available; demand reflects the extent to which the driving task consumes attention; and clarity describes participants’ comprehension and mastery of the current driving situation information. Building upon subsequent revisions from later research [43,44,45], seven supplementary dimensions were incorporated: information quantity, information quality, complexity, uncertainty, predictability, stress, and overall satisfaction. Information quantity evaluates whether the perceived amount of information in the navigation interface is appropriate; information quality reflects its accuracy, clarity, and usefulness; complexity measures the dynamism and complexity of the driving environment; uncertainty represents the driver’s confidence in the clarity of the information; predictability indicates whether participants can accurately anticipate upcoming changes; stress assesses the intensity of psychological pressure experienced during the task; and overall satisfaction reflects participants’ general evaluation of the current AR-HUD prompting strategy and interface design. Each item was rated on a 7-point Likert scale (1 = strongly disagree, 7 = strongly agree), yielding a total score range of 10–70, with higher scores indicating stronger situation awareness.

### 3.5. Experimental Procedure

Prior to the start of the experiment, informed consent forms were distributed to all 30 participants. Each participant voluntarily signed the written consent after being fully informed of the experimental background, objectives, and their related rights. Subsequently, each participant received a brief orientation to ensure a clear understanding of the experimental procedure and task requirements. Upon confirming their comprehension, participants were seated in the driving simulator, fitted with a Tobii Pro Glasses 3 wearable eye-tracking device, and completed the standard eye-tracking calibration procedure. To ensure the accuracy of data collection, participants were instructed to minimize head and body movements during the experiment and to maintain the same posture as during calibration.

**Figure 4:**
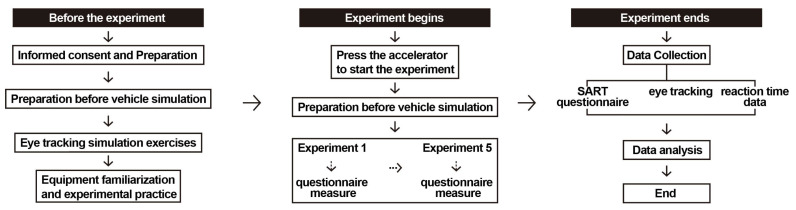
The procedure of the experiment.

Before the formal experiment began, all participants completed a practice trial to familiarize themselves with the simulated driving interface and task rhythm. During the formal experiment, participants were required to perform basic driving tasks (e.g., lane keeping, complying with speed limits) and respond to navigation prompt nodes. The experimental design included two prompt mode conditions, with each participant completing tasks under both modes in sequence and each condition containing three navigation key points. To control for extraneous variables, the experimental scenario did not include dynamic traffic elements, retaining only the road and background information to highlight the effects of multimodal prompts on attentional resource allocation. After completing each experimental task, participants immediately completed the SART questionnaire to report their subjective situation awareness under the current condition. This process was repeated until all five experimental conditions were completed. The overall experimental procedure is illustrated in Figure 4.

## 4. Results

Multimodal prompt modes and prompt timings in AR-HUD navigation had significant effects on driving behavior and subjective experience. This study systematically evaluated the effects of prompt mode (synchronous vs. asynchronous) and prompt timing (−2000 m, −1000 m, −500 m) by analyzing participants’ reaction times, average fixation duration (AFD), fixation count (FC), and subjective scores from the SART questionnaire. The SART scale encompasses seven core dimensions—information quantity, information quality, complexity, uncertainty, predictability, stress, and overall satisfaction—which collectively reflect drivers’ situation awareness under different navigation prompt strategies.

It is noteworthy that the original design included a 2 (prompt mode: synchronous vs. asynchronous) × 3 (prompt timing: −2000 m, −1000 m, −500 m) factorial structure. However, since synchronous and asynchronous stimuli at the 2000 m condition were found to be equivalent in the pilot test with no significant differences, these two were combined into a single 2000 m condition in the statistical analysis. For presentation purposes, the table still displays them as two columns, but the data have been merged and marked as duplicates (*). Accordingly, the analysis treated the within-subject factor as having five levels.

### 4.1. Reaction Time

To examine the effects of prompt mode and prompt timing on drivers’ response efficiency, the Kolmogorov–Smirnov test was first conducted on all reaction time data to assess normality. The results indicated no violation of the normal distribution assumption across groups (p>0.05), meeting the prerequisites for ANOVA. Subsequently, a two-way repeated-measures ANOVA was performed with prompt mode (synchronous vs. asynchronous) and prompt timing (−2000 m, −1000 m, −500 m) as independent variables, with reaction time as the dependent variable, systematically evaluating the impact of multimodal navigation prompts on driving performance under different conditions. Further comparisons were made on mean reaction times at different prompt timings.

As shown in Table 1, there was a significant main effect of prompt mode on reaction time, *F* (1, 174) = 16.55, p<0.001, $\eta_{p}^{2}$ = 0.09, indicating that synchronous prompts significantly reduced drivers’ reaction times. Additionally, prompt timing also showed a significant main effect, *F* (2, 174) = 5.27, p=0.006, $\eta_{p}^{2}$ = 0.06, demonstrating that prompt distance positively influenced reaction time. However, the interaction effect between prompt mode and prompt timing was not statistically significant, *F* (2, 174) = 1.11, p=0.333, $\eta_{p}^{2}$ = 0.01, suggesting that the effect of prompt mode on reaction time was consistent across different prompt timings.

**Figure 5:**
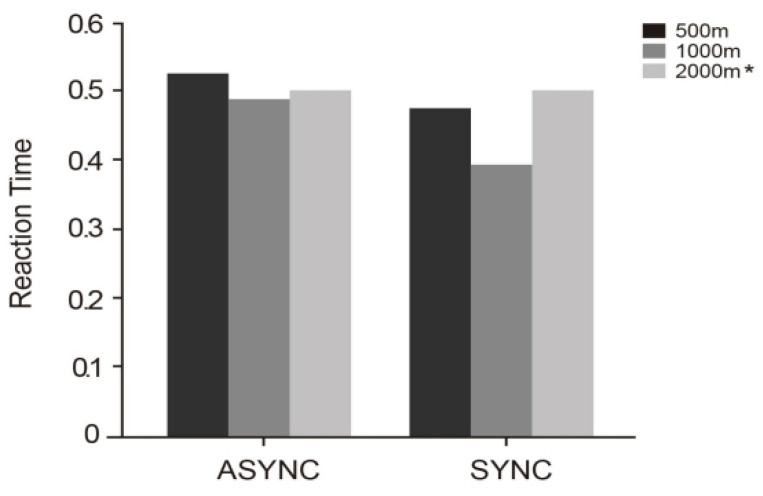
This figure shows the reaction times (in milliseconds) under five experimental conditions: SYNC-2000, SYNC-1000, SYNC-500, ASYNC-2000 *, ASYNC-1000, and ASYNC-500. The * indicates a repeated value identical to the SYNC-2000 condition.

Further inspection of the mean reaction times by specific condition combinations revealed notable differences. As shown in Table 2 and Figure 5, the SYNC-1000 condition yielded the shortest reaction time (*M* = 0.39, SD = 0.11), indicating that issuing navigation instructions synchronously at a moderate distance (1000 m) most effectively facilitates rapid driver perception and response. Following this were SYNC-500 (*M* = 0.48, SD = 0.10) and SYNC-2000 (*M* = 0.50, SD = 0.09), suggesting that synchronous mode maintains high response efficiency even when prompt timing is earlier or later. On the contrary, asynchronous conditions showed slightly decreased performance. At equal distances, ASYNC-1000 (*M* = 0.49, SD = 0.12) was significantly longer than SYNC-1000. The ASYNC-500 condition recorded the longest reaction time (*M* = 0.53, SD = 0.09), implying that the combination of asynchronous prompt mode and delayed timing may interfere with the effective allocation of attentional resources, thereby impairing rapid response to navigation cues.

Additionally, to verify the reliability and validity of the reaction time measurements, the Kaiser–Meyer–Olkin (KMO) test and Bartlett’s test of sphericity were conducted to assess sample adequacy. The results are shown in Table 3. The KMO value was 0.711, indicating a “middle” level suitable for factor analysis (KMO values between 0.7 and 0.8), which demonstrates good capability for information extraction [46]. Bartlett’s test was significant ($\chi^{2}$ (3) = 79.766, p<0.001), indicating significant correlations among variables and meeting the prerequisites for factor analysis.

Furthermore, the reliability of the reaction time measurement was assessed using Cronbach’s alpha, with the results summarized in Table 4. The overall Cronbach’s α was 0.840, exceeding the 0.8 threshold, indicating good internal consistency [47]. The corrected item-total correlations (CITCs) for each item were all above 0.4, and removing any single item did not significantly increase the α coefficient, demonstrating the stable contribution of each item to the overall reaction time measurement.

### 4.2. Eye-Tracking Data

To further investigate the effect of multimodal navigation prompts on drivers’ attention distribution, this study collected and analyzed eye-tracking data from participants during the experiment. The independent variables were prompt mode (synchronous vs. asynchronous) and prompt timing (−2000 m, −1000 m, −500 m), while the dependent variables were average fixation duration (AFD) and fixation count (FC). After confirming that the data met normality assumptions, a two-way ANOVA was performed to systematically analyze the differences in fixation behaviors across prompt conditions.

#### 4.2.1. Average Fixation Duration

As shown in Table 5, there was a significant main effect of prompt mode on average fixation duration, *F* (1, 174) = 12.39, p=0.001, $\eta_{p}^{2}$ = 0.07, indicating that synchronous prompts significantly increased drivers’ fixation stability. Additionally, prompt timing exhibited a significant main effect, *F* (2, 174) = 4.22, p=0.016, $\eta_{p}^{2}$ = 0.05, demonstrating significant differences in average fixation duration across different prompt timings. However, the interaction between prompt mode and prompt timing was not statistically significant, with results of *F* (2, 174) = 0.29, p=0.747, and $\eta_{p}^{2}\approx 0.00$.

**Figure 6:**
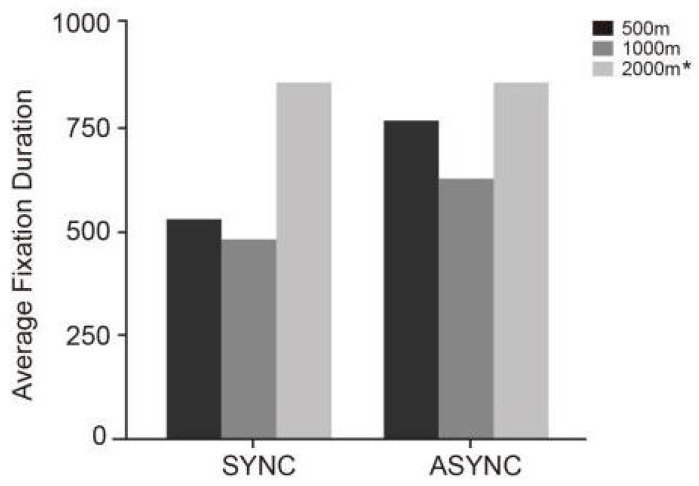
This figure illustrates the average fixation durations (in milliseconds) under six experimental conditions (SYNC-2000, SYNC-1000, SYNC-500, ASYNC-2000 *, ASYNC-1000, ASYNC-500), where “*” denotes a repeated value identical to the SYNC-2000 condition.

Further comparisons of the mean values across conditions, as shown in Table 6 and Figure 6, reveal that the average fixation duration was highest under the 2000 m prompt condition (*M* = 766.50, SD = 261.23). Within the synchronous conditions, drivers exhibited the most focused fixation under SYNC-1000 (*M* = 485.17, SD = 224.91), followed by SYNC-500 (*M* = 621.30, SD = 96.91). On the contrary, under asynchronous prompt conditions (ASYNC), drivers generally showed longer average fixation durations, with ASYNC-1000 being the shortest (*M* = 629.80, SD = 385.35) and ASYNC-500 being the longest (*M* = 861.60, SD = 556.16). Overall, fixation durations were shorter and less variable under synchronous prompting, whereas asynchronous prompting led to significantly increased fixation times, suggesting that drivers had more difficulty maintaining stable attention and exhibited more dispersed fixation behavior.

#### 4.2.2. Fixation Count

The results of the analysis are presented in Table 7. A significant main effect of prompting mode on fixation count was found, *F* (1, 174) = 23.32, p<0.001, $\eta_{p}^{2}$ = 0.12, indicating that synchronous prompts significantly increased drivers’ visual attention to the navigation area. The main effect of prompting timing was not significant, *F* (2, 174) = 0.44, p=0.643, $\eta_{p}^{2}$ = 0.01, suggesting minimal influence of prompt distance on fixation count. Additionally, the interaction between prompting mode and timing was not significant, with results of *F* (2, 174) = 0.03, p=0.974, and $\eta_{p}^{2}$ = 0.00.

**Figure 7:**
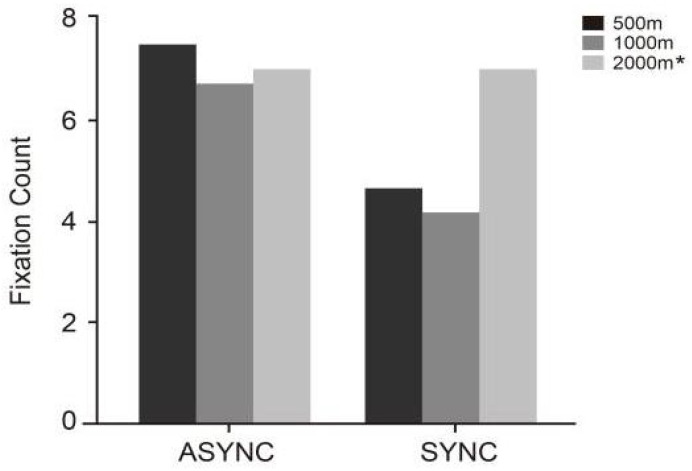
Fixation counts (unit: counts) under six experimental conditions (SYNC-2000, SYNC-1000, SYNC-500, ASYNC-2000 *, ASYNC-1000, ASYNC-500). The * indicates repeated values equal to those in the SYNC-2000 condition.

Further analysis in Table 8 and Figure 7 shows that at the 2000 m prompt distance, the fixation counts under synchronous (SYNC-2000) and asynchronous (ASYNC-2000) conditions were identical (*M* = 7.00, SD = 2.98), with both exceeding those of other synchronous conditions. Under synchronous prompting, the fixation counts were generally lower, with SYNC-1000 showing the lowest count (*M* = 4.13, SD = 2.93), followed by SYNC-500 (*M* = 4.63, SD = 2.27). This suggests that synchronous presentation helps reduce oculomotor load, enabling drivers to extract navigation information more efficiently. Conversely, the fixation counts increased significantly under asynchronous prompt conditions, with ASYNC-500 exhibiting the highest count (*M* = 7.47, SD = 5.39), followed by ASYNC-1000 (*M* = 6.70, SD = 4.13). This pattern may reflect the increased visual scanning and repeated confirmation required when prompt information is asynchronous, leading to a higher number of fixations.

### 4.3. Situation Awareness Rating Technique

The results of the analysis are presented in Table 9. A significant main effect of prompt timing on the SART scores was found, *F* (2, 174) = 60.45, p<0.001, $\eta_{p}^{2}$ = 0.41, indicating that different prompt lead times significantly influenced drivers’ sustained attention performance. The main effect of prompting mode was also significant, *F* (1, 174) = 4.59, p=0.034, $\eta_{p}^{2}$ = 0.03, suggesting that, overall, synchronous prompts slightly outperformed asynchronous ones. However, the interaction effect between prompting mode and timing was not significant, *F* (2, 174) = 0.22, p=0.805, $\eta_{p}^{2}$ = 0.002, indicating that the effect of prompting mode on sustained attention was consistent across different prompt timings.

**Figure 8:**
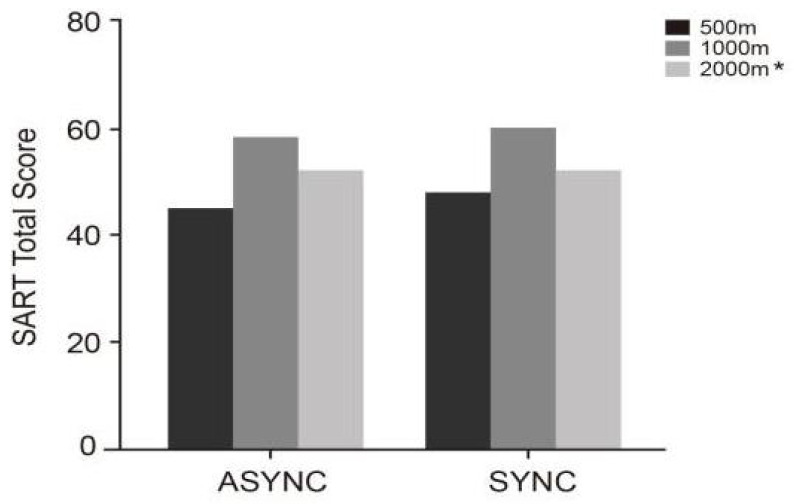
SART scores under six experimental conditions (SYNC-2000, SYNC-1000, SYNC-500, ASYNC-2000 *, ASYNC-1000, ASYNC-500). The “*” denotes repeated values equal to those in the SYNC-2000 condition.

Further comparisons in Table 10 and Figure 8 reveal that under synchronous conditions, drivers’ SART scores across the three prompt lead times were SYNC-1000 (*M* = 60.10, SD = 5.50), SYNC-2000 (*M* = 50.63, SD = 6.19), and SYNC-500 (*M* = 48.03, SD = 6.29), with SYNC-1000 performing best. This indicates that prompts presented synchronously and at a moderate distance most effectively support sustained attention. Under asynchronous conditions, a similar trend was observed, with SART scores of ASYNC-1000 (*M* = 58.50, SD = 3.81), ASYNC-2000 (*M* = 50.63, SD = 6.19), and ASYNC-500 (*M* = 45.10, SD = 9.84). Overall, regardless of prompting mode, the 1000 m condition consistently yielded the highest SART performance, underscoring the advantage of moderate prompt lead times.

## 5. Discussion

This study aimed to systematically investigate the combined effects of visual navigation and continuous voice prompts within a multimodal AR-HUD navigation system on driver behavior. The primary focus was to examine how prompting mode, timing, and their interaction influence driver reaction efficiency, attention allocation, and situation awareness in a voice navigation context.

### 5.1. Reaction Time Analysis

The results demonstrate that both the prompting mode and timing significantly affect drivers’ reaction times (p<0.001 and p=0.006), thereby supporting research questions RQ1 and RQ2. Specifically, prompting mode exerted a significant impact on operational performance, confirming RQ1. The findings reveal that synchronous prompting significantly reduced drivers’ reaction times. On the contrary, asynchronous prompts led to notably lower reaction efficiency. This indicates that when visual and auditory prompts are temporally synchronized, drivers perceive navigation information more efficiently and respond faster. This may be attributed to the increased situation awareness associated with crossmodal information processing under synchronous conditions, which optimizes the integration of information and accelerates decision making. These findings are consistent with [48], who argued that temporal alignment in multimodal integration decreases uncertainty and facilitates quicker responses.

Furthermore, RQ2 was preliminarily validated: prompts delivered at a moderate lead time of ∼1000 m optimized the balance between perception and response. Specifically, drivers exhibited the shortest reaction times at the 1000 m prompt distance (*M* = 0.39 s, SD = 0.11), outperforming both the earlier (2000 m) and later (500 m) prompt distances. This result aligns with the concept of an optimal perception–action window [49], suggesting that providing prompts within an appropriate temporal window enables drivers to translate perception into action intentions promptly, thereby minimizing cognitive delay [50].

Although the interaction effect was not statistically significant, mean comparisons indicate that the SYNC-1000 condition yielded the best performance (*M* = 0.39 s), while the ASYNC-500 condition showed the poorest (*M* = 0.53 s). This suggests that at the optimal prompt distance (∼1000 m), maintaining temporal synchrony maximizes navigation efficiency; conversely, asynchronous prompts at a closer distance to the target node (∼500 m) may disrupt ongoing cognitive processing and induce response delays. This observation aligns with the resource competition model, which posits that under information conflict or temporal misalignment, cognitive resources must frequently switch between perception and response processes, thereby impeding execution speed [51].

The primary mechanism by which synchronous prompts significantly reduce reaction time is redundancy gain. As discussed earlier, since auditory reaction time is physiologically shorter than visual RT, simultaneous presentation allows the system to be constrained by the fastest channel. The driver responds to a unified, integrated percept (Optimal Integration Model), reducing uncertainty and accelerating decision execution, thereby reducing reaction time compared to unimodal or temporally misaligned cues. This finding is highly consistent with optimal decision theories of crossmodal integration and explains how shorter reaction times in synchronous mode overcome inherent sensory processing speed differences. The SYNC-1000 condition achieved optimal performance, with a reaction time of M = 0.39 s that was significantly superior to all other combinations. This result emphasizes the critical importance of finding the optimal balance point between information timeliness and cognitive readiness (i.e., the optimal perception–action window) in navigation design.

### 5.2. Eye-Tracking Data Analysis

This study further investigated the effects of multimodal prompting on drivers’ average fixation duration (AFD) and fixation count (FC) through eye-tracking data, providing additional support for RQ1 and RQ2. The results revealed a significant main effect of prompting mode on AFD (p<0.001), with synchronous prompting conditions exhibiting notably superior AFD results compared to asynchronous conditions. In particular, the SYNC-1000 condition showed the best performance (*M* = 485.17 ms), falling within the optimal processing range of 400–600 ms. This suggests that under this condition, navigation information is presented in a concise and easily comprehensible structure, facilitating efficient visual processing and decision preparation. Conversely, asynchronous prompting conditions (e.g., ASYNC-1000) showed significantly longer fixation durations (*M* = 629.80 ms), which may reflect attention shifts away from visual cues caused by the sudden appearance of auditory prompts. Previous research indicates that under auditory interference, participants tend to underestimate visual stimuli, especially when auditory–visual cues are asynchronous and irregularly paced [52].

Moreover, at the ∼2000 m condition, the AFD (*M* = 594.87 ms) was higher than that observed in the SYNC-1000 condition. This may be due to the relatively ample lead time allowing drivers to initially acquire navigation information but then engage in additional route confirmation and situational assessment, thus extending visual dwell time. In the SYNC-500 condition, the AFD increased further (*M* = 621.30 ms), indicating that prompts given too late and too close to the intersection lead participants to repeatedly verify navigation information to ensure accuracy, thereby increasing fixation duration. Prior studies have demonstrated that drivers’ fixation durations are influenced by workload and increase with visual and cognitive processing demands [53].

Regarding fixation count, a significant main effect of prompting mode was also observed (p<0.001). The SYNC-1000 condition exhibited the lowest fixation count (*M* = 4.13), suggesting participants were able to extract information with fewer eye movements and thus achieved optimal visual efficiency. This aligns with previous findings [13,34] indicating that fewer fixations typically reflect well-designed interfaces with clear and legible information, which helps reduce perceptual. Conversely, the fixation counts significantly increased under asynchronous prompting, peaking at the ASYNC-500 condition (*M* = 7.47). This phenomenon can be explained on two levels: First, the inconsistency between auditory and visual prompts forces drivers to repeatedly shift attention and cross-check between modalities; second, a short prompt lead time near the intersection increases task urgency, inducing more frequent visual searches to reduce uncertainty [54]. Similarly, the 2000 m conditions showed elevated fixation counts (*M* = 7.00), suggesting that early prompts failed to effectively link to drivers’ immediate task goals, necessitating repeated fixations to reinforce memory [55].

### 5.3. Situation Awareness Rating Technique Analysis

This study further investigated the effects of multimodal prompting modes and prompting timing on drivers’ situation awareness. Statistical analyses showed a significant main effect of prompting timing on SART scores (p<0.001), indicating that the timing of the prompt is a key variable affecting situation awareness performance. Post hoc comparisons revealed that drivers performed best under the 1000 m prompting timing condition (synchronous: *M* = 60.10; asynchronous: *M* = 58.50), significantly outperforming the 2000 m and 500 m conditions. This suggests that a moderate prompting timing achieves a better balance between information acquisition and cognitive preparation, thus enhancing situation awareness. Conversely, prompts at 2000 m may cause attention drift or task forgetting due to early information presentation, while prompts at 500 m, close to the decision point, may sharply increase cognitive load, thereby reducing attentional stability [15].

Additionally, the main effect of prompting mode was also statistically significant (p=0.034), indicating that synchronous prompting generally leads to better situation awareness than asynchronous prompting. This supports [2], who argued that synchronous multimodal input facilitates perceptual integration, reduces crossmodal interference, and improves cognitive efficiency. Importantly, the interaction between prompting mode and prompting timing was not significant (p=0.805), suggesting that these two factors influence SART scores independently, with consistent trends across levels. Specifically, the SART scores for SYNC-1000 and ASYNC-1000 conditions were significantly higher than those for other combinations, further emphasizing the critical importance of timeliness of information in multimodal navigation prompt design.

### 5.4. Design Recommendations

Based on the results, AR-HUD navigation systems should focus on optimizing the synchrony and timing of multimodal prompts to improve drivers’ reaction speed, visual efficiency, and situation awareness. First, visual and auditory prompts must be strictly synchronized, maintaining temporal discrepancies within 100 ms. For example, the voice instruction “Turn right in 500 m” should be delivered simultaneously with the display of a right-turn arrow to reduce cognitive load and prevent response delays caused by asynchronous prompts. Regarding prompt timing, the optimal trigger distance is around 1000 m before the target, where drivers show the shortest reaction time (0.39 s), fewest fixations (4.13), and highest situation awareness score (60.10).

Therefore, a hierarchical prompting strategy is recommended: provide a brief pre-prompt at 2000 m (e.g., “keep left”), detailed synchronous multimodal instructions at 1000 m, and intensified visual prompts at 500 m (such as a color gradient shifting towards red) to alert the driver. The visual interface should present navigation information succinctly and centrally, placing key elements within a 15° field of view to enable single-glance acquisition of critical information and reduce repeated confirmation. Moreover, the system should incorporate context-adaptive features, such as shortening prompt intervals in congested or complex intersections or dynamically adjusting information density based on drivers’ fixation durations. To further enhance situation awareness, the AR-HUD can use environment-integrated displays overlaying virtual navigation cues on real lane markings and provide route previews with color gradients, helping drivers efficiently integrate navigation information with the real driving environment.

The core finding of this study—that the SYNC-1000 mode resulted in optimal performance (RT M = 0.39 s, FC M = 4.13)—can be directly applied to highly safety-critical scenarios (e.g., collision warning or sudden road hazards). In these time-constrained situations, every millisecond of reduction in reaction time is critical. Our data strongly suggest that time-critical warnings should employ strictly synchronous multimodal prompts (maintaining temporal discrepancies within 100 ms) combined with an intermediate advance timing (around 1000 m) to ensure drivers achieve a unified, immediate perception, thereby maximizing redundancy gain and minimizing potentially fatal response delays.

## 6. Conclusions

This study systematically investigated the effects of the synchrony of auditory and visual prompts (prompting mode) and the advance timing of prompts (prompting timing) on drivers’ behavioral performance and visual attention. Through a comprehensive analysis of reaction time, SART scores, and eye-tracking metrics (average fixation duration and fixation count), the results demonstrated significant effects of both prompting mode and prompting timing on driving behavior. Specifically, synchronous prompting at a moderate advance distance of −1000 m optimized drivers’ perceptual integration and response efficiency most effectively.

More concretely, synchronous prompts significantly shortened drivers’ reaction times, improved fixation stability and attentional focus, and reduced the frequency of visual scanning, indicating greater efficiency and coordination in cognitive resource allocation. On the contrary, asynchronous prompts increased the cost of information verification and processing, potentially triggering cognitive conflicts and delayed responses, especially when approaching the target point.

Although this study provides preliminary empirical support for optimizing multimodal AR-HUD navigation design, several limitations exist. First, the experiments were conducted in a simulated driving environment, which, while controlling experimental variables, cannot fully replicate the complexity of real-world traffic conditions and driving workload (e.g., the potential delay in navigation prompts that cannot be precisely controlled). Second, the participant pool primarily consisted of university students with limited driving experience, so the generalizability of findings to broader driver populations requires further validation. Third, this study focused on the synchrony of auditory and visual prompts and did not explore the potential effects of other modalities (e.g., haptic feedback) or the complexity of prompt content on driver behavior.

Future research could expand in several directions: (1) incorporating more ecologically valid real driving scenarios or on-road experiments to enhance applicability; (2) examining differential responses to multimodal prompts among various driver groups, such as older adults or novice drivers; (3) investigating the adaptability of multimodal prompts under varying driving task loads in multitask contexts; and (4) integrating physiological measures such as EEG and electrodermal activity (EDA) to further elucidate neural mechanisms underlying multimodal information integration and its dynamic regulation of cognitive load. The findings offer theoretical support and practical design guidance for multimodal information presentation and temporal coordination in future intelligent driving interfaces. Future work should also investigate the impact of non-manipulated variables, such as variations in auditory prompt intensity and pitch, and explore the use of auditory frequency gradients. Finally, comparative studies between multimodal prompts and single-mode visual or auditory stimulation are warranted to provide a more robust baseline for performance evaluation. In future studies, we plan to set upper and lower limits for driving speed to make the results more objective and representative.

## Figures and Tables

**Table 1:** Effects of prompt mode and prompt timing on reaction time.

Independent Variable	Dependent Variable	*F*	df	*p*	$\eta_{p}^{2}$
Prompt mode	Reaction time	16.55	1, 174	<0.001	0.09
Prompt timing	Reaction time	5.27	2, 174	0.006	0.06
Prompt mode × Prompt timing	Reaction time	1.11	2, 174	0.333	0.01

**Table 2:** Effects of prompt mode and timing combinations on reaction time.

Condition	SYNC-2000 (M±SD)	SYNC-1000 (M±SD)	SYNC-500b (M±SD)	ASYNC-2000 (M±SD)	ASYNC-1000 (M±SD)	ASYNC-500 (M±SD)
Reaction Time	0.50 ± 0.09	0.39 ± 0.11	0.48 ± 0.10	0.50 ± 0.09	0.49 ± 0.12	0.53 ± 0.09

**Table 3:** KMO and Bartlett’s test results.

Test Items		Value
KMO measure		0.711
Bartlett’s test	Approx. chi-square	79.766
	Degrees of freedom (df)	3
	*p*-value	<0.001

**Table 4:** Cronbach’s alpha reliability analysis results.

Item	CITC	Cronbach’s α	Cronbach’s α
−2000	0.756	0.737	0.840
−1000	0.656	0.858	
−500	0.740	0.752	

Note: Standardized Cronbach’s alpha = 0.853.

**Table 5:** Effects of prompt mode and prompt timing on average fixation duration.

Independent Variable	Dependent Variable	*F*	df	*p*	$\eta_{p}^{2}$
Prompt mode	Average fixation duration	12.39	1, 174	0.001	0.07
Prompt timing	Average fixation duration	4.22	2, 174	0.016	0.05
Prompt mode × Prompt timing	Average fixation duration	0.29	2, 174	0.747	0.00

**Table 6:** Effects of prompt mode and timing combinations on average fixation duration.

Condition	SYNC-2000 (M±SD)	SYNC-1000 (M±SD)	SYNC-500 (M±SD)	ASYNC-2000 (M±SD)	ASYNC-1000 (M±SD)	ASYNC-500 (M±SD)
Average fixation duration	766.500 ± 261.23	485.17 ± 224.91	621.30 ± 96.91	766.500 ± 261.23	629.80 ± 385.35	861.60 ± 556.16

**Table 7:** Effects of prompt mode and prompt timing on fixation count.

Independent Variable	Dependent Variable	*F*	df	*p*	$\eta_{p}^{2}$
Prompt mode	Fixation count	23.32	1, 174	0.000	0.12
Prompt timing	Fixation count	0.44	2, 174	0.643	0.01
Prompt mode × Prompt timing	Fixation count	0.03	2, 174	0.974	0.00

**Table 8:** Effects of prompt mode and timing combinations on fixation count.

Condition	SYNC-2000 (M±SD)	SYNC-1000 (M±SD)	SYNC-500 (M±SD)	ASYNC-2000 (M±SD)	ASYNC-1000 (M±SD)	ASYNC-500 (M±SD)
Fixation count	7.00 ± 2.98	4.13 ± 2.93	4.63 ± 2.27	7.00 ± 2.98	6.70 ± 4.13	7.47 ± 5.39

**Table 9:** Effects of prompt mode and prompt timing on SART.

Independent Variable	Dependent Variable	*F*	df	*p*	$\eta_{p}^{2}$
Prompt mode	Situation awareness rating technique	4.59	1, 174	0.034	0.03
Prompt timing	Situation awareness rating technique	60.45	2, 174	0.000	0.41
Prompt mode × Prompt timing	Situation awareness rating technique	0.22	2, 174	0.805	0.00

**Table 10:** Effects of prompt mode and timing combinations on SART.

Condition	SYNC-2000 (M±SD)	SYNC-1000 (M±SD)	SYNC-500 (M±SD)	ASYNC-2000 (M±SD)	ASYNC-1000 (M±SD)	ASYNC-500 (M±SD)
Situation awareness rating technique	50.63 ± 6.19	60.10 ± 5.50	48.03 ± 6.29	50.63 ± 6.19	58.50 ± 3.81	45.10 ± 9.84

## Data Availability

The datasets used during the current study are available from the corresponding author upon reasonable request.

## Abbreviations

The following abbreviations are used in this manuscript:

SYNC	Synchronous
ASYNC	Asynchronous
SART	Situation awareness rating technique
AFD	Average fixation duration
FC	Fixation count
